# Baseline *TREM-1* Whole Blood Gene Expression Does Not Predict Response to Adalimumab Treatment in Patients with Ulcerative Colitis or Crohn’s Disease in the SERENE Studies

**DOI:** 10.1093/ecco-jcc/jjad170

**Published:** 2023-10-06

**Authors:** Bram Verstockt, Valerie Pivorunas, Naim Al Mahi, Nizar Smaoui, Heath Guay, Nicholas A Kennedy, James R Goodhand, Simeng Lin, Benjamin Y H Bai, Stephen B Hanauer, Marc Ferrante, Julian Panés, Séverine Vermeire

**Affiliations:** Department of Gastroenterology and Hepatology, University Hospitals Leuven, Leuven, Belgium; Department of Chronic Diseases and Metabolism, KU Leuven, Leuven, Belgium; Precision Medicine Immunology, AbbVie Bioresearch Centre, Worcester, MA, USA; Genomic Research Center, AbbVie, North Chicago, IL, USA; Genomic Research Center, AbbVie, North Chicago, IL, USA; Precision Medicine Immunology, AbbVie Bioresearch Centre, Worcester, MA, USA; Department of Gastroenterology, Royal Devon University Healthcare NHS Foundation Trust, Exeter, UK; Department of Gastroenterology, Royal Devon University Healthcare NHS Foundation Trust, Exeter, UK; Department of Gastroenterology, Royal Devon University Healthcare NHS Foundation Trust, Exeter, UK; Genomics of Inflammation and Immunity Group, Wellcome Sanger Institute, Hinxton, UK; Department of Medicine, Northwestern University Feinberg School of Medicine, Evanston, IL, USA; Department of Gastroenterology and Hepatology, University Hospitals Leuven, Leuven, Belgium; Department of Chronic Diseases and Metabolism, KU Leuven, Leuven, Belgium; Hospital Clinic de Barcelona, IDIBAPS, CIBERehd, Barcelona, Catalonia, Spain; Department of Gastroenterology and Hepatology, University Hospitals Leuven, Leuven, Belgium; Department of Chronic Diseases and Metabolism, KU Leuven, Leuven, Belgium

**Keywords:** Biomarkers, clinical trials

## Abstract

**Background and Aims:**

This study assessed whether baseline triggering receptor expressed on myeloid cells [*TREM-1*] whole blood gene expression predicts response to anti-tumour necrosis factor [anti-TNF] therapy in patients with ulcerative colitis [UC] or Crohn’s disease [CD].

**Methods:**

*TREM-1* whole blood gene expression was analysed by RNA sequencing in patients with moderately to severely active UC or CD treated with adalimumab in the Phase 3 SERENE-UC and SERENE-CD clinical trials. The predictive value of baseline *TREM-1* expression was evaluated and compared according to endoscopic and clinical response vs non-response, and remission vs non-remission, at Weeks 8 and 52 [SERENE-UC], and Weeks 12 and 56 [SERENE-CD].

**Results:**

*TREM-1* expression was analysed in 95 and 106 patients with UC and CD, respectively, receiving standard-dose adalimumab induction treatment. In SERENE-UC, baseline *TREM-1* expression was not predictive of endoscopic response [*p* = 0.48], endoscopic remission [*p* = 0.53], clinical response [*p* = 0.58], or clinical remission [*p* = 0.79] at Week 8, or clinical response [*p* = 0.60] at Week 52. However, an association was observed with endoscopic response [*p* = 0.01], endoscopic remission [*p* = 0.048], and clinical remission [*p* = 0.04997] at Week 52. For SERENE-CD, baseline *TREM-1* expression was not predictive of endoscopic response [*p* = 0.56], endoscopic remission [*p* = 0.33], clinical response [*p* = 0.07], or clinical remission [*p* = 0.65] at Week 12, or endoscopic response [*p* = 0.61], endoscopic remission [*p* = 0.51], clinical response [*p* = 0.62], or clinical remission [*p* = 0.97] at Week 56.

**Conclusions:**

Baseline *TREM-1* gene expression did not uniformly predict adalimumab response in SERENE clinical trials. Further research is needed to identify potential blood-based biomarkers predictive of response to anti-TNF therapy in patients with inflammatory bowel disease.

**Clinicaltrials.gov identifiers:**

NCT02065622; NCT02065570

## 1. Introduction

Ulcerative colitis [UC] and Crohn’s disease [CD] are immune-mediated inflammatory bowel diseases [IBDs] with a complex interplay of genetic and environmental factors involved in their pathogenesis.^1^ Adalimumab is an anti-tumour necrosis factor [anti-TNF] agent that is approved by the US Food and Drug Administration^2^ and the European Medicines Agency^3^ for the treatment of moderately to severely active UC and CD. Although anti-TNF agents such as adalimumab play an important role in the management of IBD, primary non-response to anti-TNFs occurs in 10–40% of patients.^4^ Identifying factors that predict response to anti-TNF therapy may improve the success of the treat-to-target approach recommended in IBD management guidelines.^5,6^

Conflicting evidence exists regarding whether baseline triggering receptor expressed on myeloid cells [*TREM-1*] whole blood gene expression level is predictive of response to anti-TNF agents in patients with UC or CD.^7–11^ In one study, baseline whole blood *TREM-1* expression was downregulated in a mixed population of patients with UC or CD who achieved endoscopic remission following anti-TNF treatment, and low gut mucosa and whole blood expression of *TREM-1* demonstrated similar accuracy as biomarkers of anti-TNF responsiveness; in contrast, a low serum *TREM-1* protein level was not predictive of response.^7^ Another study showed that low baseline whole blood *TREM-1* levels could be used as a potential predictive biomarker of anti-TNF therapeutic efficacy, in terms of mucosal healing in patients with CD.^8^ Consistent with these results, further analysis found an association between high *TREM-1* expression levels in cluster of differentiation 14-positive monocytes and decreased differentiation to M2-type regulatory macrophages, which are involved in mediating the anti-TNF response, indicating that response to anti-TNF agents may be linked to low *TREM-1* levels in monocytes in patients with IBD.^9^ In contrast to these findings, significantly downregulated baseline *TREM-1* whole blood expression was predictive for non-response to anti-TNF therapy in a small cohort of patients with CD, based on clinical and/or endoscopic improvement of IBD-related symptom response criteria.^10^ Gene expression data from a cell-centred meta-analysis conducted by the authors of this latter study suggested that, in individuals who do not respond to anti-TNF agents, an increase in inflammatory macrophages is associated with upregulation of *TREM-1* and chemokine receptor type 2-chemokine ligand 7 axes.^10^ Further, results from a study of paediatric patients with IBD suggested that baseline *TREM-1* whole blood expression was not predictive for response to anti-TNF therapy, with no differences seen between responders and non-responders.^11^ One potential explanation for these opposing findings is the widespread use of corticosteroid [CS] drugs among patients with IBD.^12^

The effect of CS use on *TREM-1* expression is unclear and may be dependent on the individual CS. For example, in murine models, the CS dexamethasone has been shown to suppress *TREM*-*1* expression on neutrophils, with this suppressive effect being mediated by TNF-alpha; in contrast, hydrocortisone had no effect on *TREM-1* expression compared with controls.^13^ The differences in study designs, patient populations and their CS use, and findings mean that the potential utility of baseline *TREM-1* whole blood gene expression as a predictive biomarker for response to anti-TNF therapy has yet to be elucidated.

The aim of the current study was to determine whether baseline *TREM-1* whole blood gene expression was predictive of outcomes following standard adalimumab treatment in patients with UC or CD in the large, randomized, Phase 3 SERENE-UC and -CD studies.^14,15^

## 2. Methods

### 2.1. SERENE-UC and SERENE-CD study designs

SERENE-UC [NCT02065622]^14^ and SERENE-CD [NCT02065570]^15^ were Phase 3, double-blind, randomized, multicentre clinical trials that evaluated higher vs standard adalimumab induction dosing regimens [Table 1] followed by adalimumab maintenance therapy in adult patients with moderately to severely active UC [*n* = 852] or CD [*n* = 514]. The primary endpoint of SERENE-UC was clinical remission as per full Mayo score at Weeks 8 and 52. The co-primary endpoints of SERENE-CD were Clinical Disease Activity Index clinical remission at Week 4 and endoscopic response at Week 12. The full methods and results of SERENE-UC^14^ and SERENE-CD^15^ have been published previously.

**Table 1.** Adalimumab induction dosing regimens in SERENE-UC and SERENE-CD.

	SERENE-UC^14^	SERENE-CD^15^
Higher dose	160 mg at Weeks 0, 1, 2, and 3, followed by 40 mg at Weeks 4 and 6	160 mg at Weeks 0, 1, 2, and 3, followed by 40 mg every other week from Weeks 4 to 12
Standard dose	160 mg at Week 0 followed by 80 mg at Week 2 and 40 mg at Weeks 4 and 6	160 mg at Week 0 followed by 80 mg at Week 2 and 40 mg every other week from Weeks 4 to 12

CD, Crohn’s disease; UC, ulcerative colitis.

### 2.2. Assessment of clinical outcomes

In the present study, clinical and endoscopic response and remission, defined in Table 2, were assessed at Weeks 8 and 52 in the SERENE-UC population, and at Weeks 12 and 56 in SERENE-CD participants.

**Table 2.** Definitions of clinical and endoscopic response and remission used in the analysis of data from SERENE-UC and SERENE-CD, and in previously published studies of the ability of baseline whole blood *TREM-1* expression to predict response to anti-TNF treatment in patients with IBD.

Endpoint	SERENE-UC		SERENE-CD		Verstockt et al.^7^		Prins et al.^9^	Gaujoux et al.^10^
	Week 8	Week 52	Week 12	Week 56	UC	CD	CD	UC/CD
					Week 8/14	6 months	6 months	Week ≥14
Clinical response	Full Mayo score decrease of ≥3 points and ≥30% from baseline, plus a ≥1-point decrease in rectal bleeding subscore or an absolute bleeding score of ≤1	Full Mayo score decrease of ≥3 points and ≥30% from baseline, plus a ≥1-point decrease in rectal bleeding subscore or an absolute bleeding score of ≤1 in Week 8 responders	≥70-point decrease in CDAI score	≥70-point decrease in CDAI score	—	—	—	Physician-defined clinical and/or endoscopic improvement of IBD symptoms plus a decision to continue anti-TNF therapy
Clinical remission	Full Mayo score ≤2 with no subscore >1	Full Mayo score ≤2 with no subscore >1 in Week 8 remitters	CDAI <150	CDAI <150	—	—	—	—
Endoscopic response^a^	Endoscopic subscore ≤1	Endoscopic subscore ≤1 in Week 8 responders	>50% decrease from baseline in SES-CD [or a ≥2-point reduction in patients with a baseline SES-CD of 4]	>50% decrease from baseline in SES-CD [or a ≥2-point reduction in patients with a baseline SES-CD of 4]	—	—	—	—
Endoscopic remission	Endoscopic subscore 0	Endoscopic subscore 0 in Week 8 remitters	SES-CD ≤4 plus ≥2-point decrease from baseline and no subscore >1 in any individual variable	SES-CD ≤4 plus ≥2-point decrease from baseline and no subscore >1 in any individual variable	Endoscopic subscore ≤1	SES-CD ≤2	Complete absence of ulcerations	—

^a^Described as ‘endoscopic improvement’ in the SERENE-UC study.^14^

CD, Crohn’s disease; CDAI, Clinical Disease Activity Index; IBD, inflammatory bowel disease; SES-CD, Simple Endoscopic Score for Crohn’s Disease; TNF, tumour necrosis factor; *TREM-1*, triggering receptor expressed on myeloid cells; UC, ulcerative colitis.

### 2.3. Evaluation of *TREM-1* expression

In patients who provided consent for exploratory biomarker analyses, whole blood *TREM-1* gene expression was assessed by RNA sequencing [RNA-seq] at baseline and Weeks 2, 4, and 8 for UC, and at baseline and Weeks 2, 4, and 12 for CD. Single-end RNA-seq was performed to generate 76-bp reads. The samples were enriched by poly-A selection and globulin depletion with 14 Universal Human Reference RNA controls in 24 sequencing flow cells. Unique molecular identifier tagging was also used to improve sensitivity. Average unique read numbers were 20.6 million [standard error (SE): 5.04 million] in UC and 15.2 million [SE: 7.2 million] in CD. Soluble TREM-1 protein levels at baseline were also assessed using the Olink inflammation panel.

### 2.4. Statistical considerations

As this was a post hoc analysis, the sample sizes for each analysis were not pre-specified, and were instead determined by the numbers of patients from each trial who provided consent for exploratory molecular biomarker analyses. Unless otherwise specified, analyses were conducted only in the subset of patients who received standard-dose induction adalimumab. A univariate multiple regression analysis corrected for multiple testing with baseline *TREM-1* gene expression [log_2_ counts per million] as the response and baseline CS use as a predictor was performed using data from each trial separately to assess whether CS use impacted *TREM-1* gene expression.

Baseline *TREM-1* gene and soluble protein expression were analysed in patients evaluated for clinical outcomes at Weeks 8 and 52 in SERENE-UC, and at Weeks 12 and 56 in SERENE-CD. Two-sample t-tests were used to compare:

- Baseline *TREM-1* gene and soluble protein expression in clinical remitters vs clinical non-remitters, clinical responders vs clinical non-responders, endoscopic remitters vs endoscopic non-remitters, and endoscopic responders vs endoscopic non-responders. Definitions of all these endpoints are provided in Table 2.
- Change in *TREM-1* gene expression from baseline to Weeks 2, 4, and 8 for UC, and from baseline to Weeks 2, 4, and 12 for CD.

A logistic regression model was fitted to calculate area under the curve [AUC] values for baseline *TREM-1* gene expression for clinical remission and response, and endoscopic remission and response at Weeks 8 and 52 [SERENE-UC] and Weeks 12 and 56 [SERENE-CD].

In addition, baseline *TREM-1* gene expression was compared with a two-sample t-test in clinical and endoscopic remitters and responders vs non-remitters and non-responders stratified into quartiles based on Week 4 adalimumab levels [25%: 0‒2.5 mg/mL; 50%: 2.6‒5.4 mg/mL; 75%: 5.5‒8.8 mg/mL; 100%: 8.9‒33.2 mg/mL]. Adalimumab levels were measured using an enzyme-linked immunosorbent assay in SERENE-UC^14^ and a validated ligand-binding assay in SERENE-CD.^15^ The drug level stratification analyses included patients who received the higher dose of adalimumab, as well as those treated with the standard dose in SERENE-UC and SERENE-CD, to capture and assess data from patients with the widest possible range of drug exposure levels. Data for all these analyses are presented as box plots of the median [interquartile range], while the *p*-values were calculated using the baseline mean [standard deviation]. All *p*-values were adjusted for multiple testing correction using the Benjamini–Hochberg method.^16^ To evaluate whether the results of this analysis were impacted by non-responders with inadequate drug levels, this analysis was repeated in only patients receiving the standard or high dose of adalimumab with adequate drug levels [>5 µg/mL] at Week 8 [SERENE-UC] and Week 12 [SERENE-CD].

The relationships between baseline *TREM-1* gene expression and change from baseline levels, and neutrophil percentage as a proportion of the total leucocyte population, neutrophil absolute counts, and faecal calprotectin were assessed, grouped by endoscopic outcomes, and statistically tested using Pearson’s correlation. Neutrophil percentage analyses were also performed stratified by prior CS use at baseline, given the possible impact of CS use on *TREM-1* expression.^13^ The neutrophil analyses were performed using complete blood count levels, with neutrophils chosen because of their relatively high expression of *TREM-1* compared with other leucocytes. All *p*-values reported throughout are nominal. Patients or the public were not involved in the design, conduct, reporting, or dissemination plans of our research.

### 2.5. Ethical statement

The independent ethics committee or institutional review board at each study site approved the study protocol, informed consent forms, recruitment materials, and all other patient information related to both SERENE studies. Examples of ethics committees/institutional review boards for both studies include Advarra Inc. [formerly Quorum Review Inc., USA], Helsinki Committee [Israel], Western University Health Science Research Ethic Board [Canada], and Comitato Etico IRCCS [Italy]. All approvals were obtained before study drug shipment to a site; however, approval reference numbers for each site are not available because the sponsor’s standard operating procedure is to archive studies within 1 year of completion of the clinical study report. All patients in both studies provided written informed consent ahead of screening; informed consent was also provided by patients for optional testing [i.e. by the subset of patients whose samples were used for the exploratory biomarker analyses that are presented here].

## 3. Results

In SERENE-UC, whole blood *TREM-1* gene expression was analysed by RNA-seq in 95 and 70 patients who were receiving the standard adalimumab dose at Weeks 8 and 52, respectively. In SERENE-CD, *TREM-1* gene expression was analysed in 106 patients who were receiving the standard dose at Week 12, and in 50 and 48 patients for endoscopic and clinical outcomes, respectively, at Week 56 [clinical data were not available at Week 56 for two patients]. Patient demographics and baseline characteristics are reported for the *TREM-1* subsets and the overall study cohorts by endoscopic and clinical response/non-response and remission/non-remission outcomes in SERENE-UC [Weeks 8 and 52; Supplementary Tables 1 and 2] and SERENE-CD [Weeks 12 and 56; Supplementary Tables 3 and 4]. The baseline demographics and clinical characteristics of both the UC and CD patient subsets analysed by endoscopic and clinical outcomes were generally representative of the overall study cohorts that have been reported previously.^14,15^ An exception to this was noted in SERENE-UC, where across all endpoints analysed, the proportion of male patients was higher in the overall population than in the RNA-seq subgroup [Supplementary Tables 1 and 2]. Additionally, in SERENE-CD, a higher proportion of RNA-seq patients reported aminosalicylate use vs the overall population, and Clinical Disease Activity Index scores were slightly lower in the RNA-seq subgroup vs the overall population across all endpoints analysed [Supplementary Tables 3 and 4]. Importantly, all 95 [100%] patients in the SERENE-UC *TREM-1* subset were White; in SERENE-CD, 91.8% of the 106 patients were White, 6.4% were Black or African American, and 1.8% were Asian.

Finally, baseline CS use was associated with significantly lower baseline *TREM-1* gene expression in SERENE-UC [*p* = 0.008], but not in SERENE-CD [*p* > 0.05].

### 3.1. Baseline *TREM-1* expression levels by endoscopic and clinical outcomes

**Figure 1.**
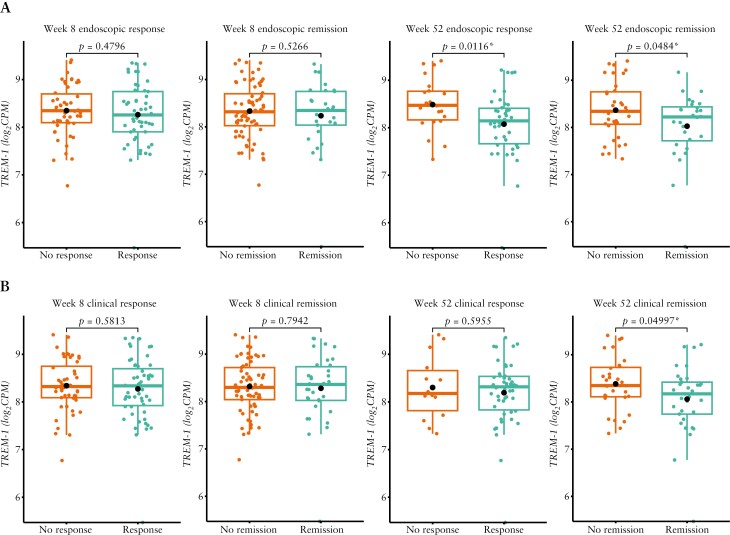
Baseline *TREM-1* whole blood expression in patients with UC treated with the standard dose of adalimumab in SERENE-UC who had [A] endoscopic response/non-response and remission/non-remission at Weeks 8 and 52 and [B] clinical response/non-response and remission/non-remission at Weeks 8 and 52.^a,b^ Data are median [interquartile range], with the *p*-values calculated using the baseline mean [standard deviation]. ^a^Definitions of endoscopic and clinical outcomes are listed in Table 2. ^b^*n* = 95 at Week 8; *n* = 70 at Week 52. *p < 0.05. *TREM-1*, triggering receptor expressed on myeloid cells; UC, ulcerative colitis.

Among patients with UC who received the standard adalimumab dose, baseline *TREM-1* gene expression did not differ in those who achieved endoscopic response at Week 8 vs non-responders at this time point; the same was true of endoscopic remitters vs non-remitters at Week 8 [Figure 1A]. Significant but minor differences were observed based on endoscopic response [*p* = 0.0116] and endoscopic remission [*p* = 0.0484] at Week 52 [Figure 1A]. Regarding clinical outcomes, baseline *TREM-1* gene expression did not differ between patients with UC who achieved clinical response vs non-response at Week 8 or 52, or between clinical remitters vs non-remitters at Week 8; however, there was a significant but minor difference for clinical remission at Week 52 [*p* = 0.05; Figure 1B]. The AUC values in patients with UC were low and ranged from 0.48 to 0.69.

In patients with UC, baseline soluble TREM-1 protein levels did not differ in endoscopic or clinical remitters or responders at Week 8 or 52 vs non-remitters and non-responders, respectively [*p* > 0.05].

**Figure 2.**
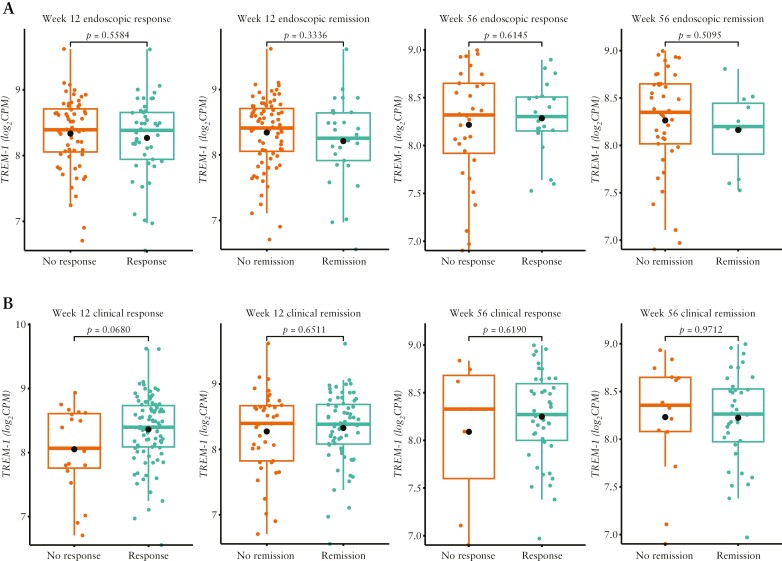
Baseline *TREM-1* whole blood expression in patients with CD treated with the standard dose of adalimumab in SERENE-CD who had [A] endoscopic response/non-response and remission/non-remission at Weeks 12 and 56 and [B] clinical response/non-response and remission/non-remission at Weeks 12 and 56.^a,b^ Data are median [interquartile range], with the *p*-values calculated using the baseline mean [standard deviation]. ^a^Definitions of endoscopic and clinical outcomes are listed in Table 2. ^b^*n* = 106 at Week 12; *n* = 50 and *n* = 48 at Week 56 for endoscopic and clinical outcomes, respectively. CD, Crohn’s disease; *TREM-1*, triggering receptor expressed on myeloid cells.

In patients with CD, baseline *TREM-1* gene expression did not differ in endoscopic remitters or responders at Week 12 or 56 vs non-remitters and non-responders, respectively, at these time points [Figure 2A]. As with the UC population, there was also no significant difference in baseline *TREM-1* gene expression in patients with CD who were clinical remitters vs non-remitters, or clinical responders vs non-responders, at either evaluation time point following standard adalimumab dosing [Figure 2B]. The AUC values in patients with CD were low and ranged from 0.48 to 0.63.

In patients with CD, baseline soluble TREM-1 protein levels did not differ in endoscopic remitters or responders at Week 12 or 56 vs non-remitters and non-responders, or clinical remitters at Week 56 vs non-remitters [all *p* > 0.05]; however, there was a nominal difference between clinical responders and non-responders at Week 56 [*p* = 0.04].

**Figure 3.**
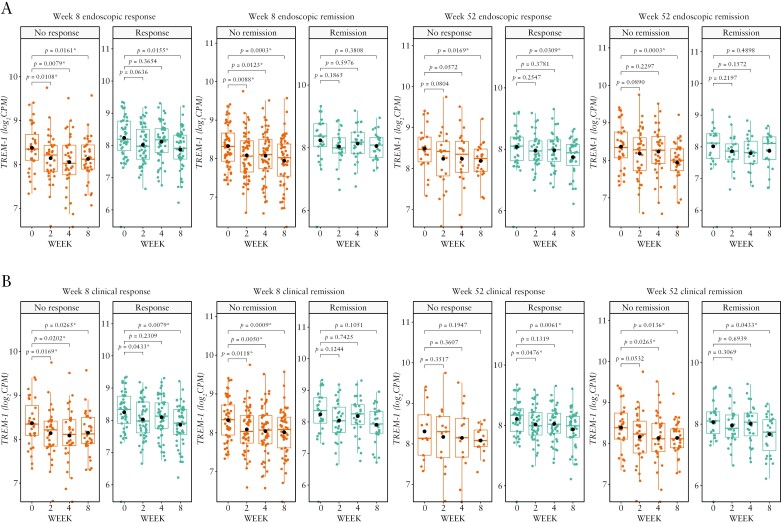
*TREM-1* whole blood expression over time in patients with UC treated with the standard dose of adalimumab in SERENE-UC who had [A] endoscopic response/non-response and remission/non-remission at Weeks 8 and 52 and [B] clinical response/non-response and remission/non-remission at Weeks 8 and 52.^a,b^ Data are median [interquartile range], with the *p*-values calculated using the baseline mean [standard deviation]. ^a^Definitions of endoscopic and clinical outcomes are listed in Table 2. ^b^*n* = 95 at Week 8; *n* = 70 at Week 52. *p < 0.05. *TREM-1,* triggering receptor expressed on myeloid cells; UC, ulcerative colitis.

**Figure 4.**
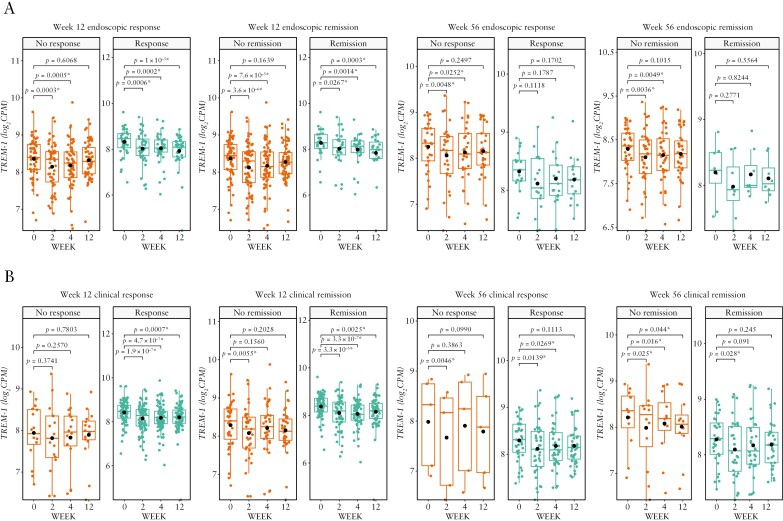
*TREM-1* whole blood expression over time in patients with CD treated with the standard dose of adalimumab in SERENE-CD who had [A] endoscopic response/non-response and remission/non-remission at Weeks 12 and 56 and [B] clinical response/non-response and remission/non-remission at Weeks 12 and 56.^a,b^ Data are median [interquartile range], with the *p*-values calculated using the baseline mean [standard deviation]. ^a^Definitions of endoscopic and clinical outcomes are listed in Table 2. ^b^*n* = 106 at Week 12; *n* = 50 and *n* = 48 at Week 56 for endoscopic and clinical outcomes, respectively. *p < 0.05. CD, Crohn’s disease; *TREM-1*, triggering receptor expressed on myeloid cells.

Regardless of endoscopic or clinical outcome at any time point, *TREM-1* gene expression generally decreased from baseline over time in both studies [Figures 3 and 4]. In SERENE-UC, the decrease was particularly marked in endoscopic and clinical non-remitters [*p* < 0.001 at Week 8 among patients who were endoscopic or clinical non-remitters at Week 8 or endoscopic non-remitters at Week 52, and *p* < 0.05 at Week 8 among clinical non-remitters at Week 52; Figure 3]. By contrast, in SERENE-CD, the greatest decreases from baseline in *TREM-1* gene expression were seen in endoscopic and clinical remitters and responders [*p* < 0.001 at Week 12 among patients who were endoscopic or clinical responders, or endoscopic remitters, and *p* < 0.01 among patients who were clinical remitters at Week 12; Figure 4].

Baseline *TREM-1* gene expression was not predictive of endoscopic outcomes at Week 8 or 52 when patients with UC receiving either the high [*n* = 152 at Week 8 and *n* = 113 at Week 52] or the standard [*n* = 95 and *n* = 70, respectively] dose of adalimumab were stratified by Week 4 per-quartile drug levels [Supplementary Figure 1]. Similarly, baseline *TREM-1* gene expression was not predictive of endoscopic outcomes at Week 12 or 56 in SERENE-CD when stratified by per-quartile drug levels in patients receiving the high [*n* = 150 at Week 12 and *n* = 61 at Week 56] or standard [*n* = 106 and *n* = 50, respectively] dose of adalimumab [Supplementary Figure 2]. Regardless of adalimumab dose, baseline *TREM-1* gene expression was not predictive of clinical outcomes at Week 8 or 52 in patients with UC [Supplementary Figure 3], or in patients with CD at Week 12 or 56 [Supplementary Figure 4], when stratified by per-quartile drug levels. Similar results were found when the analysis was repeated only in patients with adequate adalimumab levels [>5 µg/mL] at Week 8 [SERENE-UC] and Week 12 [SERENE-CD]. Baseline *TREM-1* gene expression did not predict endoscopic outcomes at Week 8 or 52 in patients with UC, or Week 12 or 56 in patients with CD. For clinical outcomes, baseline *TREM-1* gene expression was not predictive of clinical response at Week 8 or 52 in patients with UC, or clinical remission at Week 8; however, unlike the Week 4 drug level analysis, it was predictive of clinical remission at Week 52. For patients with CD, baseline *TREM-1* gene expression was not predictive of clinical response or remission at Week 12 or 56 [data not shown].

### 3.2. Association of baseline *TREM-1* gene expression with baseline neutrophil percentage and faecal calprotectin grouped by endoscopic outcomes

**Figure 5.**
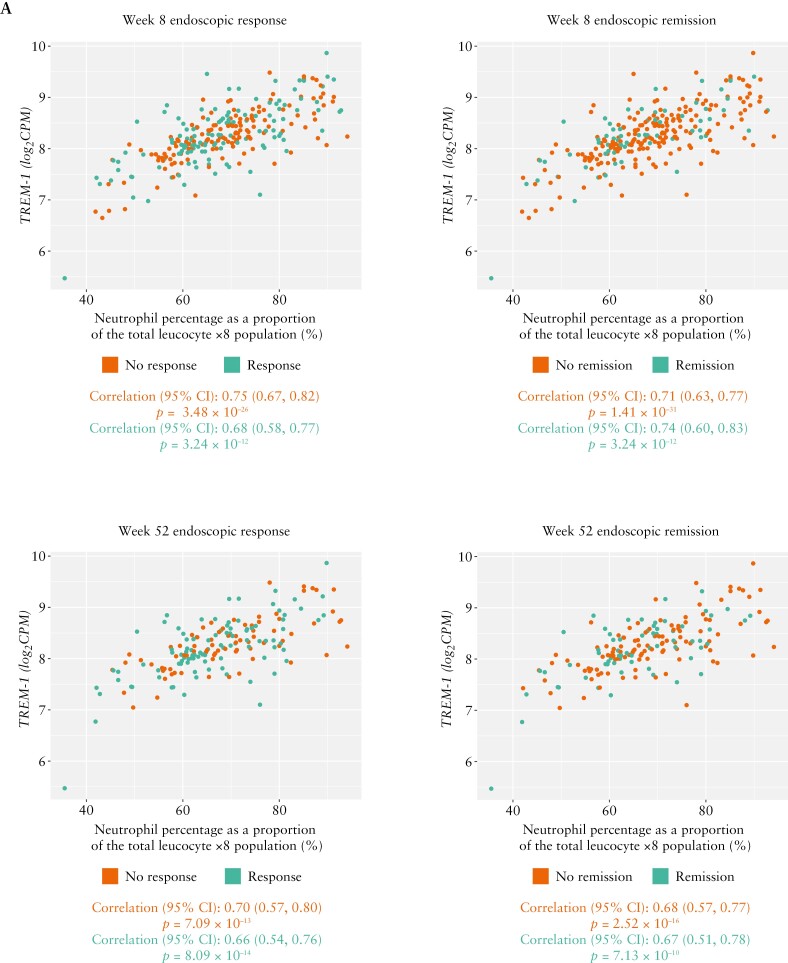

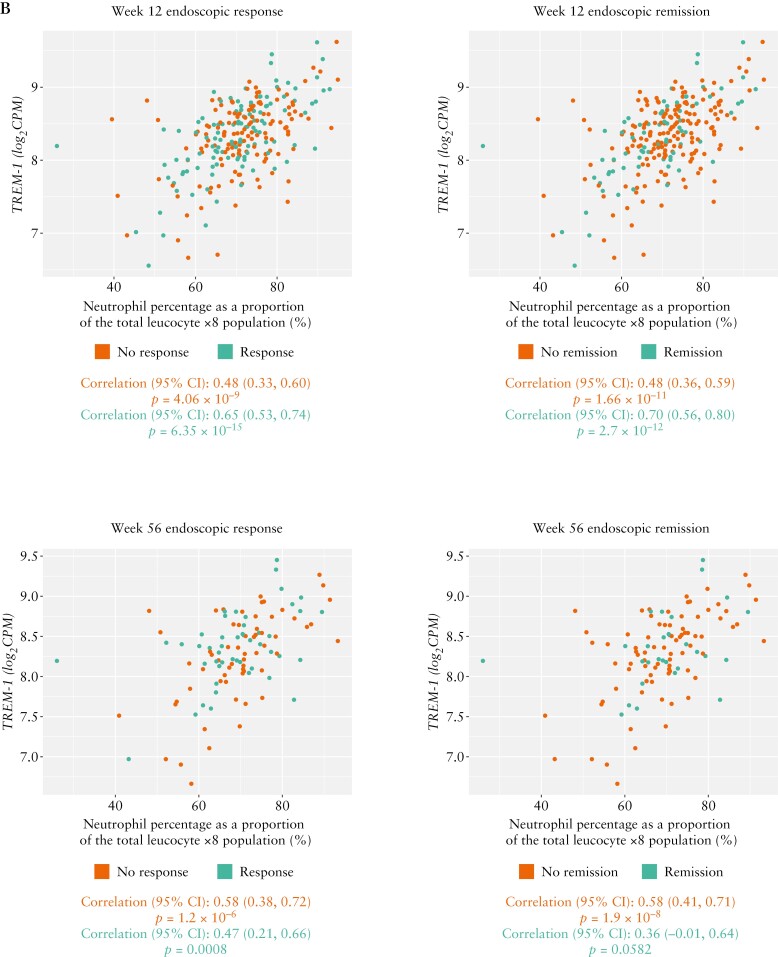
Association of baseline *TREM-1* whole blood expression with baseline neutrophil percentage by endoscopic response/non-response and remission/non-remission^a^ in patients with UC and CD treated with the standard dose of adalimumab in [A] SERENE-UC and [B] SERENE-CD. All correlations were statistically significant [*p* < 0.05]. ^a^Definitions of endoscopic outcomes are listed in Table 2. CD, Crohn’s disease; CI, confidence interval; *TREM-1*, triggering receptor expressed on myeloid cells; UC, ulcerative colitis.

In patients treated with standard-dose adalimumab, baseline neutrophil percentage was highly positively correlated with baseline *TREM-1* gene expression in responders and non-responders, and remitters and non-remitters across endoscopic outcomes in patients with UC [Week 8: *r* = 0.68 for response vs *r* = 0.75 for non-response, and *r* = 0.74 for remitters vs *r* = 0.71 for non-remitters; Week 52: *r* = 0.66 for response vs *r* = 0.70 for non-response, and *r* = 0.67 for remitters vs *r* = 0.68 for non-remitters; Figure 5A] and CD [Week 12: *r* = 0.65 for response vs *r* = 0.48 for non-response, and *r* = 0.70 for remitters vs *r* = 0.48 for non-remitters; Week 56: *r* = 0.47 for response vs *r* = 0.58 for non-response, and *r* = 0.36 for remitters vs *r* = 0.58 for non-remitters; Figure 5B]. All correlations were significant [*p* < 0.001] except for remitters at Week 56 in SERENE-CD [*p* = 0.06]. No notable differences were observed between remitters vs non-remitters or responders vs non-responders. Similar results were seen when stratified further by patients with and without steroid exposure history, with no notable differences between groups. In addition, similar patterns were seen when analysing the relationships between absolute neutrophil counts instead of percentages and baseline *TREM-1* expression, as well as change from baseline *TREM-1* expression and neutrophil percentage [data not shown].

**Figure 6.**
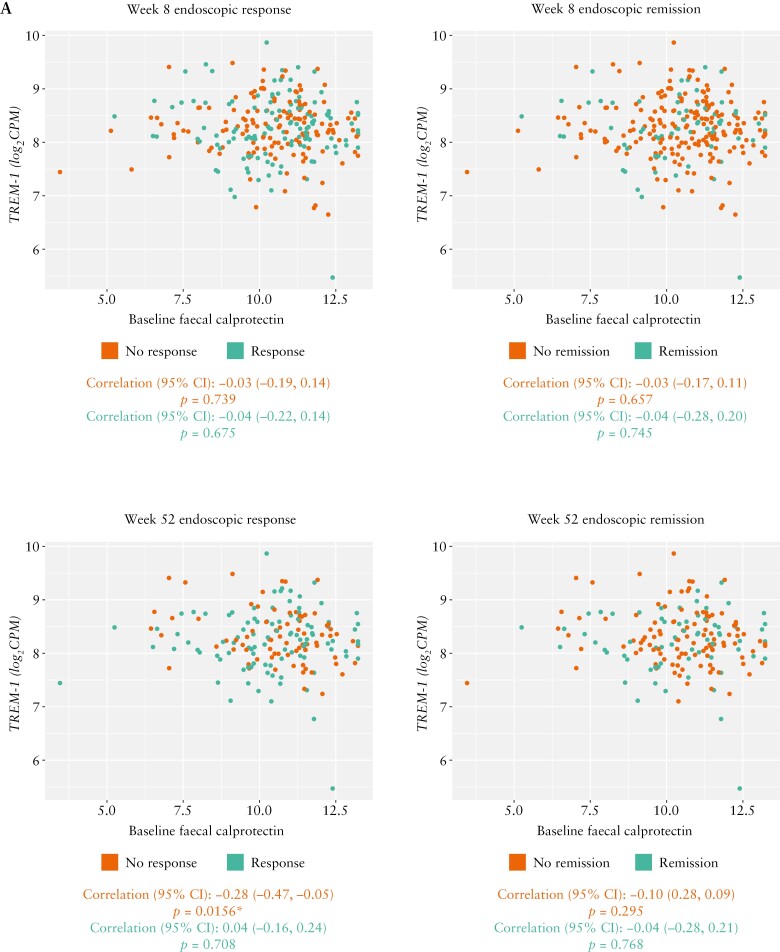

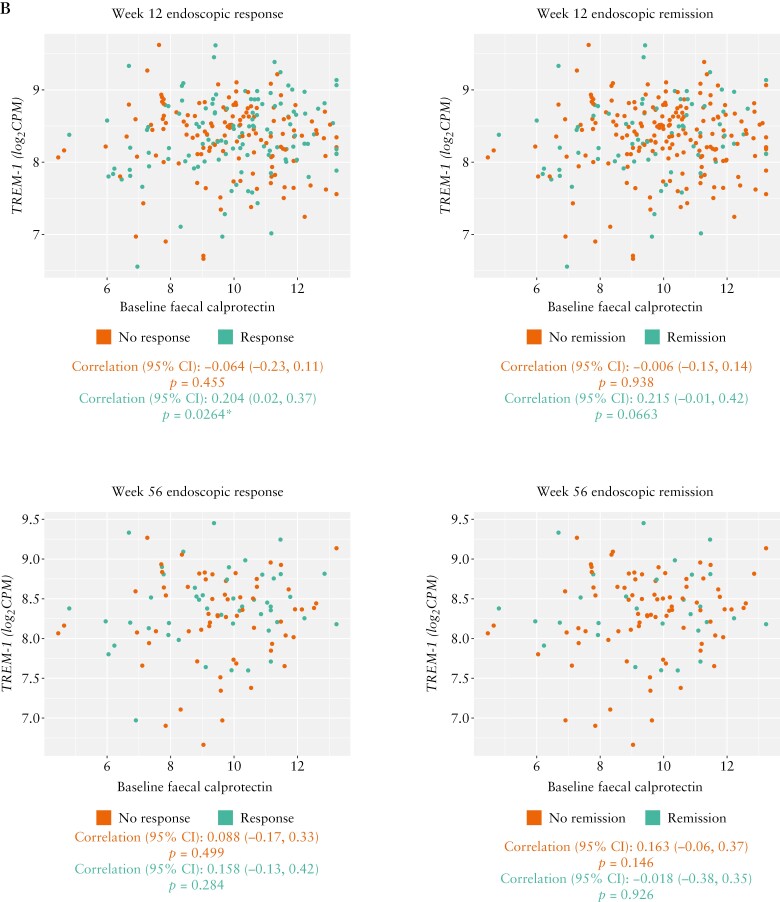
Association of baseline *TREM-1* whole blood expression with faecal calprotectin by endoscopic response/non-response and remission/non-remission^a^ in patients with UC and CD treated with the standard dose of adalimumab in [A] SERENE-UC and [B] SERENE-CD. ^ a^Definitions of endoscopic outcomes are listed in Table 2. *p < 0.05. CD, Crohn’s disease; CI, confidence interval; *TREM-1*, triggering receptor expressed on myeloid cells; UC, ulcerative colitis.

In contrast to the association seen with neutrophils, no strong association was observed between faecal calprotectin levels and baseline *TREM-1* gene expression for any endoscopic outcome in patients with either UC [Week 8: *r* = −0.04 for both responders and remitters vs *r* = −0.03 for both non-responders and non-remitters; Week 52: *r* = 0.04 for responders vs *r* = −0.28 for non-responders, and *r* = −0.04 for remitters vs *r* = −0.10 for non-remitters; Figure 6A] or CD [Week 12: *r* = 0.20 for responders vs *r* = −0.06 for non-responders, and *r* = 0.215 for remitters vs *r* = −0.006 for non-remitters; Week 56: *r* = 0.16 for responders vs *r* = 0.09 for non-responders, and *r* = −0.02 for remitters vs *r* = 0.16 for non-remitters; Figure 6B]. All correlations were non-significant [*p* > 0.05], except for endoscopic non-responders at Week 52 in SERENE-UC [*p* = 0.02] and endoscopic responders at Week 12 [*p* = 0.03] in SERENE-CD. Similar results were seen for the relationships between change from baseline in faecal calprotectin levels and baseline *TREM-1* expression [data not shown].

## 4. Discussion

The findings of this study demonstrate that baseline *TREM-1* whole blood gene expression was not a predictor of clinical or endoscopic outcomes following adalimumab treatment in the Phase 3 SERENE studies in patients with moderately to severely active UC or CD. In SERENE-UC, baseline *TREM-1* gene expression was weakly associated with endoscopic response and remission after 52 weeks of adalimumab treatment, but this was not sufficiently robust for *TREM-1* to be considered a predictive biomarker of response to adalimumab. Similarly, baseline *TREM-1* gene expression did not predict clinical outcomes in SERENE-UC, or clinical or endoscopic outcomes in SERENE-CD. Largely similar results were observed when analysed using TREM-1 soluble protein levels. Stratification by per-quartile drug levels did not increase the ability of baseline *TREM-1* gene expression to predict outcomes [including when only patients with adalimumab levels >5 µg/mL were analysed], suggesting that these results are not influenced by adalimumab dose.

The results of the current study are consistent with those of both the Personalising Anti-TNF Therapy in Crohn’s disease study of patients with CD [NCT03088449]^17^ and a small study in 33 children with IBD, in which baseline *TREM-1* whole blood gene expression did not predict response to either adalimumab or infliximab.^11^ However, they contrast with the findings of other studies where *TREM-1* appeared to be predictive of clinical and/or endoscopic outcomes in patients with IBD, albeit with opposing signals for low vs high baseline *TREM-1* expression levels linked to response.^7,8,10^

The discrepancy between the results of our study and those of previous studies may be attributable to variations in study designs and patient populations. For example, in the current study, samples were analysed from a relatively large number of patients from the SERENE randomized trials [95 and 106 patients with UC and CD, respectively]. In contrast, previous studies analysed smaller samples of patients with IBD [54,^7^ 22,^10^ and 33^11^ patients], generally treated in real-world clinical practice settings. Additionally, the methods used to measure whole blood *TREM-1* gene expression differed between studies: we used RNA-seq to measure the full transcript, whereas real-time reverse-transcription polymerase chain reaction was used in the studies by Verstockt and Salvador-Martin, with the full transcript and three *TREM-1* isoforms being studied by Verstockt et al. [*TREM-1-*mb, *TREM-1*-sv, and *TREM-1*-x2].^7,11^ Finally, the definitions of clinical and endoscopic outcomes and the timing of assessments differed between studies [Table 2].

Regardless of clinical or endoscopic remission/non-remission and response/non-response, *TREM-1* gene expression generally decreased over time following adalimumab treatment in both SERENE-UC and SERENE-CD. This suggests that *TREM-1* could be a marker of anti-TNF treatment. Indeed, the link between downregulation of *TREM-1* and response was specific to patients treated with anti-TNF agents in one of the studies by Verstockt et al.^7^ It is possible that treatment of IBD with anti-TNF agents could reduce signalling through receptors such as *TREM-1* and lead to general downregulation, even if this does not result in clinical or endoscopic response or remission.

Of note, a highly positive correlation was observed between baseline *TREM-1* gene expression and the baseline percentage of neutrophils in both endoscopic remitters/non-remitters and responders/non-responders in both SERENE-UC [Weeks 8 and 52] and SERENE-CD [Weeks 12 and 56], except for remitters at Week 56 in SERENE-CD. This correlation was unaffected by steroid treatment history, and supports previous reports of *TREM-1* activation modulating the function of neutrophils in models of IBD.^18^ While faecal calprotectin has been related to anti-TNF response and is usually monitored in clinical practice,^19,20^ we did not find a strong correlation between *TREM-1* expression and absolute baseline or change from baseline in faecal calprotectin levels. This is consistent with the findings of Verstockt et al.^7^

Our analysis was limited in that it focused solely on *TREM-1* expression, which has been suggested previously as a potential blood-based biomarker for anti-TNF response. Further analyses, using an unbiased, genome-wide approach as well as genetic studies at the individual level, may allow for identification of novel predictive biomarkers in whole blood and at the cellular level. However, findings generated from such a genome-wide approach would need confirmation in independent cohorts. Another limitation is that all patients in the subset of patients analysed from SERENE-UC and 92% of patients from SERENE-CD were White; this limited diversity means that the analysis may not be generalizable to other racial groups. Finally, dichotomous [response vs non-response]rather than continuous outcomes were analysed, which can lead to a loss of power for detecting a difference between groups; however, these outcomes were based on protocol-defined primary and secondary endpoints used for the primary analysis of each trial.

In summary, the results of this robust analysis in patients with moderately to severely active UC or CD from the large, randomized, Phase 3 SERENE studies demonstrate that baseline *TREM-1* whole blood gene expression does not predict endoscopic or clinical outcomes following adalimumab treatment. Consequently, further exploration of potential predictors of response to anti-TNF therapy is needed to identify non-invasive biomarkers that could be used in clinical practice to allow personalized treatment and optimize treat-to-target management of IBD.

## Supplementary Material

jjad170_suppl_Supplementary_Materials

## Data Availability

The data underlying this article cannot be shared publicly due to patient consent and privacy sharing limitations.

## Conference presentation

Part of this work was presented at the 17th Congress of the European Crohn’s and Colitis Organisation, 16–19 February 2022, Vienna, Austria.
