# Supplementary material for: Baseline TREM-1 Whole Blood Gene Expression Does Not Predict Response to Adalimumab Treatment in Patients with Ulcerative Colitis or Crohn’s Disease in the SERENE Studies
Source: J Crohns Colitis. 2023 Oct 6;18(4):493–505. doi: 10.1093/ecco-jcc/jjad170 (PMC11037103; doi:10.1093/ecco-jcc/jjad170)
Supplement: jjad170_suppl_Supplementary_Materials [file jjad170_suppl_supplementary_materials.docx]

**Supplementary Table 1.** Baseline demographics and clinical characteristics by endoscopic outcomes in the overall population and the subset of patients analysed for *TREM-1* expression in SERENE-UC.

| **Characteristic** | **Week 8 endoscopic responders** | | **Week 8 endoscopic non-responders** | | | **Week 52 endoscopic responders** | | **Week 52 endoscopic non-responders** | | | **Week 8 endoscopic remitters** | | | **Week 8 endoscopic non-remitters** | | **Week 52 endoscopic remitters** | | **Week 52 endoscopic non-remitters** | | |
| --- | --- | --- | --- | --- | --- | --- | --- | --- | --- | --- | --- | --- | --- | --- | --- | --- | --- | --- | --- | --- |
|  | ***TREM-1*  subset**  **[*n* = 133]** | **Overall population**  **[*n* = 286]** | | ***TREM-1* subset**  **[*n* = 146]** | **Overall population**  **[*n* = 666]** | ***TREM-1* subset**  **[*n* = 104]** | **Overall**  **population**  **[*n* = 319]** | ***TREM-1* subset**  **[*n* = 81]** | **Overall population**  **[*n* = 316]** | ***TREM-1* subset**  **[*n* = 70]** | | **Overall population**  **[*n* = 116]** | ***TREM-1* subset [*n* = 209]** | | **Overall population [*n* = 836]** | ***TREM-1* subset [*n* = 71]** | **Overall population [*n* = 197]** | | ***TREM-1* subset [*n* = 114]** | **Overall population [*n* = 438]** |
| **Male sex, n [%]** | 56  [42.1] | 145*  [50.7] | | 64  [43.8] | 402*  [60.4] | 46  [44.2] | 181*  [56.7] | 36  [44.4] | 191*  [60.4] | 26  [37.1] | | 56*  [48.3] | 94  [45.0] | | 491*  [58.7] | 29  [40.8] | 108*  [54.8] | | 53  [46.5] | 264*  [60.3] |
| **Age, years [range]** | 38.7  [18–75] | 38.8 [18–75] | | 39.5  [18–73] | 41.1 [18–74] | 39.9  [18–75] | 40.6 [18–75] | 38.8  [18–65] | 40.2 [18–71] | 35.8  [18–67] | | 36.4 [18–67] | 40.2  [18–75] | | 40.9 [18–75] | 37.9  [18–75] | 39.8 [18–75] | | 40.4  [18–70] | 40.7 [18–71] |
| **Disease duration, years** | 6.8  [6.7] | 7.3  [6.9] | | 7.9  [8.2] | 7.2  [7.2] | 7.2  [7.8] | 7.3  [7.5] | 6.1  [5.4] | 6.5  [6.3] | 7.3  [7.5] | | 7.1  [7.4] | 7.4  [7.5] | | 7.2  [7.1] | 6.8  [7.3] | 7.0  [7.5] | | 6.6  [6.6] | 6.8  [6.7] |
| **BMI, kg/m^2^** | 24.7  [5.3] | 25.0  [5.3] | | 24.6  [5.2] | 25.2  [5.3] | 24.7  [4.8] | 25.5  [5.4] | 24.8  [5.3] | 25.0  [5.0] | 24.1  [5.4] | | 24.5  [5.7] | 24.9  [5.2] | | 25.2  [5.3] | 24.2  [4.9] | 25.2  [5.5] | | 25.0  [5.1] | 25.3  [5.1] |
| **Corticosteroid use,  n [%]** | 96  [72.2] | 193  [67.5] | | 79  [54.1] | 375  [56.3] | 73  [70.2] | 203  [63.6] | 47  [58.0] | 196  [62.0] | 51  [72.9] | | 76  [65.5] | 124  [59.3] | | 492  [58.9] | 50  [70.4] | 127  [64.5] | | 70  [61.4] | 272  [62.1] |
| **Immunosuppressant use, n [%]** | 30  [22.6] | 83  [29.0] | | 36  [24.7] | 185  [27.8] | 24  [23.1] | 101  [31.7] | 23  [28.4] | 97  [30.7] | 17  [24.3] | | 29  [25.0] | 49  [23.4] | | 239  [28.6] | 16  [22.5] | 64  [32.5] | | 31  [27.2] | 134  [30.6] |
| **Aminosalicylate use,  n [%]** | 102  [76.7] | 226  [79.0] | | 113  [77.4] | 515  [77.3] | 82  [78.8] | 255  [79.9] | 64  [79.0] | 259  [82.0] | 56  [80.0] | | 94  [81.0] | 159  [76.1] | | 647  [77.4] | 56  [78.9] | 159  [80.7] | | 90  [78.9] | 355  [81.1] |
| **Prior infliximab use,  n [%]** | 7  [5.3] | 25  [8.7] | | 24  [16.4] | 110  [16.5] | 9  [8.7] | 33  [10.3] | 10  [12.3] | 44  [13.9] | 3  [4.3] | | 7  [6.0] | 28  [13.4] | | 128  [15.3] | 6  [8.5] | 20  [10.2] | | 13  [11.4] | 57  [13.0] |
| **Full Mayo score** | 8.5  [1.5] | 8.3  [1.5] | | 8.9  [1.5] | 9.0  [1.5] | 8.5  [1.5] | 8.6  [1.6] | 8.8  [1.5] | 8.8  [1.5] | 8.2  [1.4] | | 8.2  [1.4] | 8.9  [1.5] | | 8.9  [1.5] | 8.5  [1.5] | 8.6  [1.6] | | 8.7  [1.5] | 8.7  [1.5] |
| **Adapted Mayo score** | 6.3  [1.4] | 6.1  [1.4] | | 6.5  [1.3] | 6.7  [1.3] | 6.3  [1.5] | 6.3  [1.5] | 6.5  [1.3] | 6.5  [1.2] | 6.0  [1.4] | | 5.9  [1.4] | 6.6  [1.3] | | 6.6  [1.3] | 6.3  [1.5] | 6.4  [1.5] | | 6.5  [1.3] | 6.5  [1.3] |
| **Partial Mayo score** | 6.3  [1.4] | 6.1  [1.4] | | 6.5  [1.4] | 6.6  [1.4] | 6.3  [1.4] | 6.3  [1.5] | 6.4  [1.3] | 6.4  [1.3] | 6.0  [1.3] | | 5.9  [1.4] | 6.5  [1.4] | | 6.5  [1.4] | 6.3  [1.5] | 6.4  [1.6] | | 6.4  [1.3] | 6.3  [1.4] |
| **Serum albumin,  mg/mL** | 40.4  [4.1] | 40.6  [3.8] | | 39.9  [4.5] | 39.6  [4.2] | 40.4  [3.6] | 40.2  [3.5] | 39.4  [4.5] | 39.8  [4.0] | 40.8  [3.7] | | 40.8  [4.0] | 40.0  [4.5] | | 39.8  [4.1] | 41.0  [3.5] | 40.6  [3.6] | | 39.3  [4.2] | 39.8  [3.8] |
| **Faecal calprotectin,  µg/g** | 2391.4 [2334.7] | 2499.4 [2547.7] | | 2288.2 [2202.1] | 2555.3 [2568.8] | 2287.6  [2396.7] | 2405.7  [2507.8] | 2432.4  [2073.1] | 2500.9  [2410.3] | 2277.0  [2353.8] | | 2522.0  [2658.5] | 2356.5  [2234.9] | | 2541.0  [2549.4] | 2421.7  [2545.6] | 2426.6  [2474.8] | | 2308.7 [2070.5] | 2465.1 [2453.4] |
| **hs-CRP, mg/L** | 10.7  [18.9] | 8.9  [15.1] | | 11.2  [16.3] | 12.8  [18.0] | 10.6  [19.7] | 10.1  [15.5] | 13.2  [15.5] | 12.4  [16.8] | 9.1  [10.5] | | 9.4  [12.6] | 11.6  [19.3] | | 11.9  [17.8] | 7.5  [8.9] | 8.6  [10.6] | | 14.3  [21.4] | 12.5  [18.0] |
| **Neutrophils, %** | 67.5  [11.7] | 68.7  [11.6] | | 69.0  [11.4] | 68.2  [11.7] | 66.4  [11.2] | 67.8  [11.0] | 69.5  [12.0] | 68.7  [12.2] | 66.2  [12.1] | | 67.0  [12.3] | 69.0  [11.3] | | 68.5  [11.6] | 65.5  [11.2] | 67.1  [10.9] | | 69.1  [11.7] | 68.8  [11.9] |

All data are mean [standard deviation] unless otherwise specified.

**p* <0.05 vs *TREM-1* subset.

BMI, body mass index; hs-CRP, high-sensitivity C-reactive protein; *TREM-1*, triggering receptor expressed on myeloid cells; UC, ulcerative colitis.

**Supplementary Table 2.** Baseline demographics and clinical characteristics by clinical outcomes in the overall population and the subset of patients analysed for *TREM-1* expression in SERENE-UC.

| **Characteristic** | **Week 8 clinical responders** | | **Week 8 clinical  non-responders** | | | **Week 52 clinical responders** | | **Week 52 clinical non-responders** | | | **Week 8 clinical remitters** | | | **Week 8 clinical non-remitters** | | **Week 52 clinical remitters** | | **Week 52 clinical non-remitters** | | |
| --- | --- | --- | --- | --- | --- | --- | --- | --- | --- | --- | --- | --- | --- | --- | --- | --- | --- | --- | --- | --- |
|  | ***TREM-1*  subset**  **[*n* = 143]** | **Overall population**  **[*n* = 430]** | | ***TREM-1* subset**  **[*n* = 136]** | **Overall population**  **[*n* = 522]** | ***TREM-1* subset**  **[*n* = 132]** | **Overall**  **population**  **[*n* = 426]** | ***TREM-1* subset**  **[*n* = 53]** | **Overall population**  **[*n* = 209]** | ***TREM-1* subset**  **[*n* = 87]** | | **Overall population**  **[*n* = 127]** | ***TREM-1* subset**  **[*n* = 192]** | | **Overall population**  **[*n* = 825]** | ***TREM-1* subset**  **[*n* = 83]** | **Overall population**  **[*n* = 236]** | | ***TREM-1* subset**  **[*n* = 102]** | **Overall population**  **[*n* = 399]** |
| **Male sex, n [%]** | 62  [43.4] | 241*  [56.0] | | 58  [42.6] | 306*  [58.6] | 60  [45.5] | 250*  [58.7] | 22  [41.5] | 122*  [58.4] | 40  [46.0] | | 67*  [52.8] | 80  [41.7] | | 480*  [58.2] | 37  [44.6] | 133*  [56.4] | | 45  [44.1] | 239*  [59.9] |
| **Age, years [range]** | 38.2  [18–75] | 39.5 [18–75] | | 40.0 [18–73] | 41.2 [18–73] | 39.8  [18–75] | 40.7 [18–75] | 38.3  [18–62] | 39.7 [18–71] | 36.9  [18–71] | | 37.6 [18–71] | 40.1  [18–75] | | 40.8 [18–75] | 39.5  [18–75] | 40.1 [18–75] | | 39.3  [18–68] | 40.6 [18–71] |
| **Disease duration, years** | 7.2  [7.3] | 7.3  [7.1] | | 7.6  [7.7] | 7.1  [7.1] | 7.2  [7.4] | 7.1  [7.1] | 5.5  [5.2] | 6.5  [6.6] | 7.5  [7.2] | | 7.7  [7.0] | 7.3  [7.6] | | 7.1  [7.1] | 7.8  [8.2] | 7.4  [7.6] | | 5.8  [5.3] | 6.6  [6.5] |
| **BMI, kg/m^2^** | 24.7  [5.2] | 25.0  [5.2] | | 24.7  [5.4] | 25.3  [5.5] | 25.0  [4.9] | 25.4  [5.3] | 24.2  [5.4] | 25.0  [5.1] | 23.9  [4.9] | | 24.2  [4.7] | 25.1  [5.4] | | 25.3  [5.4] | 24.7  [5.0] | 25.2  [5.2] | | 24.8  [5.1] | 25.3  [5.2] |
| **Corticosteroid use,  n [%]** | 99  [69.2] | 275  [64.0] | | 76  [55.9] | 293  [56.1] | 90  [68.2] | 277  [65.0] | 30  [56.6] | 122  [58.4] | 64  [73.6] | | 90  [70.9] | 111  [57.8] | | 478  [57.9] | 58  [69.9] | 153  [64.8] | | 62  [60.8] | 246  [61.7] |
| **Immunosuppressant use, n [%]** | 34  [23.8] | 128  [29.8] | | 32  [23.5] | 140  [26.8] | 31  [23.5] | 133  [31.2] | 16  [30.2] | 65  [31.1] | 21  [24.1] | | 33  [26.0] | 45  [23.4] | | 235  [28.5] | 21  [25.3] | 81  [34.3] | | 26  [25.5] | 117  [29.3] |
| **Aminosalicylate use,  n [%]** | 107  [74.8] | 341  [79.3] | | 108  [79.4] | 400  [76.6] | 105  [79.5] | 350  [82.2] | 41  [77.4] | 164  [78.5] | 64  [73.6] | | 96  [75.6] | 151  [78.6] | | 645  [78.2] | 63  [75.9] | 185 [78.4] | | 83  [81.4] | 329  [82.5] |
| **Prior infliximab use,  n [%]** | 8  [5.6] | 47  [10.9] | | 23  [16.9] | 88  [16.9] | 12  [9.1] | 49  [11.5] | 7  [13.2] | 28  [13.4] | 5  [5.7] | | 11  [8.7] | 26  [13.5] | | 124  [15.0] | 8  [9.6] | 28  [11.9] | | 11  [10.8] | 49  [12.3] |
| **Full Mayo score** | 8.7  [1.6] | 8.7  [1.6] | | 8.7  [1.5] | 8.8  [1.5] | 8.7  [1.5] | 8.8 [1.6] | 8.4  [1.4] | 8.5  [1.4] | 8.3  [1.5] | | 8.3  [1.6] | 8.9  [1.5] | | 8.9  [1.5] | 8.6  [1.5] | 8.5  [1.7] | | 8.7  [1.5] | 8.8 [1.5] |
| **Adapted Mayo score** | 6.4  [1.4] | 6.5  [1.4] | | 6.4  [1.3] | 6.6  [1.3] | 6.5  [1.5] | 6.5  [1.4] | 6.2  [1.2] | 6.3  [1.3] | 6.1  [1.5] | | 6.0  [1.5] | 6.6  [1.3] | | 6.6  [1.3] | 6.3  [1.5] | 6.3  [1.6] | | 6.4  [1.3] | 6.5  [1.3] |
| **Partial Mayo score** | 6.5  [1.4] | 6.4  [1.5] | | 6.4  [1.4] | 6.4  [1.4] | 6.4  [1.4] | 6.5  [1.5] | 6.1  [1.2] | 6.1 [1.3] | 6.1  [1.4] | | 6.1  [1.5] | 6.5  [1.4] | | 6.5  [1.4] | 6.3  [1.4] | 6.2  [1.6] | | 6.4  [1.4] | 6.4  [1.3] |
| **Serum albumin,  mg/mL** | 40.3  [4.1] | 40.3  [4.0] | | 40  [4.6] | 39.6  [4.2] | 40  [4.0] | 40.2  [3.8] | 39.9  [4.1] | 39.7  [3.7] | 41.0  [3.8] | | 40.8  [4.1] | 39.8  [4.5] | | 39.7  [4.1] | 40.4  [3.5] | 40.3  [3.6] | | 39.6  [4.4] | 39.8  [3.8] |
| **Faecal calprotectin,  µg/g** | 2424.5 [2343.0] | 2631.7 [2646.6] | | 2247.6 [2181.2] | 2463.6 [2490.4] | 2366.8 [2316.8] | 2538.5 [2519.7] | 2312.5 [2114.2] | 2278.6 [2322.6] | 2334.9 [2338.2] | | 2818.2 [2780.3] | 2337.1 [2231.8] | | 2496.4 [2525.6] | 2317.8 [2350.0] | 2467.9 [2525.2] | | 2377.6 [2189.1] | 2444.9 [2421.7] |
| **hs-CRP, mg/L** | 11.0  [18.6] | 11.1  [17.5] | | 10.9  [16.4] | 12.0  [17.1] | 11.8  [19.4] | 11.3  [17.0] | 11.4  [13.9] | 11.1  [14.5] | 9.6  [12.2] | | 9.5  [12.7] | 11.6  [19.5] | | 12.0  [17.9] | 11.6  [21.6] | 11.3  [17.4] | | 11.8  [14.5] | 11.2  [15.5] |
| **Neutrophils, %** | 67.8  [11.9] | 68.8  [11.9] | | 68.7  [11.2] | 68.0  [11.5] | 67.2  [11.5] | 68.1  [11.0] | 68.9  [11.8] | 68.6  [12.8] | 67.6  [11.9] | | 68.4  [12.8] | 68.6  [11.5] | | 68.4  [11.5] | 66.4  [11.4] | 67.7  [11.2] | | 68.8  [11.7] | 68.6  [11.9] |

All data are mean [standard deviation] unless otherwise specified.

**p* <0.05 vs *TREM-1* subset.

BMI, body mass index; hs-CRP, high-sensitivity C-reactive protein; *TREM-1*, triggering receptor expressed on myeloid cells; UC, ulcerative colitis.

**Supplementary Table 3.** Baseline demographics and clinical characteristics by endoscopic outcomes in the overall population and the subset of patients analysed for *TREM-1* expression in SERENE-CD.

| **Characteristic** | **Week 12 endoscopic responders** | | **Week 12 endoscopic non-responders** | | | **Week 56 endoscopic responders** | | | **Week 56 endoscopic non-responders** | | **Week 12 endoscopic remitters** | | | **Week 12 endoscopic non-remitters** | | **Week 56 endoscopic remitters** | | **Week 56 endoscopic non-remitters** | | |
| --- | --- | --- | --- | --- | --- | --- | --- | --- | --- | --- | --- | --- | --- | --- | --- | --- | --- | --- | --- | --- |
|  | ***TREM-1*  subset**  **[*n* = 124]** | **Overall population**  **[*n* = 213]** | | ***TREM-1* subset**  **[*n* = 142]** | **Overall population**  **[*n* = 301]** | ***TREM-1* subset**  **[*n* = 52]** | **Overall**  **population**  **[*n* = 90]** | ***TREM-1* subset**  **[*n* = 64]** | | **Overall population**  **[*n* = 128]** | ***TREM-1* subset**  **[*n* = 78]** | **Overall population**  **[*n* = 142]** | ***TREM-1* subset**  **[*n* = 188]** | | **Overall population**  **[*n* = 372]** | ***TREM-1* subset**  **[*n* = 31]** | **Overall population**  **[*n* = 63]** | | ***TREM-1* subset**  **[*n* = 85]** | **Overall population**  **[*n* = 155]** |
| **Male sex, n [%]** | 52  [41.9] | 91  [42.7] | | 69  [48.6] | 156  [51.8] | 24  [46.2] | 44  [48.9] | 31  [48.4] | | 66  [51.6] | 32  [41.0] | 60  [42.3] | 89  [47.3] | | 187  [50.3] | 15  [48.4] | 32  [50.8] | | 40  [47.1] | 78  [50.3] |
| **Age, years [range]** | 35.4  [18–69] | 36.0  [18–71] | | 37.1  [18–73] | 36.6 [18–73] | 35.0 [18–69] | 33.8  [18–69] | 35.9 [18–73] | | 36.7 [18–73] | 35.0 [18–69] | 36.2 [18–71] | 36.9 [18–73] | | 36.5 [18–73] | 37.4  [18–69] | 34.9  [18–69] | | 34.8  [18–73] | 35.8  [18–73] |
| **Disease duration, years** | 6.1  [6.4] | 6.6  [7.2] | | 6.3  [7.7] | 7.8  [9.3] | 5.2  [6.0] | 4.9  [5.6] | 6.4  [7.7] | | 7.5  [9.1] | 6.0  [6.8] | 6.9  [8.0] | 6.4  [7.3] | | 7.5  [8.7] | 5.0  [7.0] | 4.4  [6.0] | | 6.2  [7.0] | 7.3  [8.5] |
| **BMI, kg/m^2^** | 24.4  [5.8] | 24.5  [5.8] | | 24.9  [5.9] | 25.5  [6.3] | 23.7  [5.1] | 23.6  [5.3] | 24.5  [4.9] | | 25.4  [5.9] | 25.1  [6.3] | 24.8  [5.8] | 24.4  [5.6] | | 25.2  [6.2] | 24.4  [5.6] | 24.3  [5.6] | | 24.0  [4.8] | 24.8  [5.8] |
| **Corticosteroid use,  n [%]** | 71  [57.3] | 109  [51.2] | | 75  [52.8] | 146  [48.5] | 32  [61.5] | 54  [60.0] | 32  [50.0] | | 58  [45.3] | 47  [60.3] | 77 [54.2] | 99  [52.7] | | 178  [47.8] | 19  [61.3] | 39  [61.9] | | 45  [52.9] | 73  [47.1] |
| **Immunosuppressant use, n [%]** | 36  [29.0] | 56  [26.3] | | 29  [20.4] | 83  [27.6] | 15  [28.8] | 22  [24.4] | 21  [32.8] | | 40  [31.2] | 19  [24.4] | 28  [19.7] | 46  [24.5] | | 111  [29.8] | 9  [29.0] | 15  [23.8] | | 27  [31.8] | 47  [30.3] |
| **Aminosalicylate use,  n [%]** | 59  [47.6] | 87  [40.8] | | 52  [36.6] | 95  [31.6] | 27  [51.9] | 41  [45.6] | 31*  [48.4] | | 44  [34.4] | 40  [51.3] | 62  [43.7] | 71  [37.8] | | 120  [32.3] | 16 [51.6] | 29  [46.0] | | 42*  [49.4] | 56  [36.1] |
| **Prior infliximab use,  n [%]** | 16  [12.9] | 28  [13.1] | | 29  [20.4] | 61  [20.3] | 6  [11.5] | 7  [7.8] | 11  [17.2] | | 23  [18.0] | 7  [9.0] | 19  [13.4] | 38  [20.2] | | 70  [18.8] | 2  [6.5] | 3  [4.8] | | 15  [17.6] | 27  [17.4] |
| **Serum albumin,  mg/mL** | 40.1  [4.3] | 40.3  [4.1] | | 39.5  [4.2] | 39.6  [4.0] | 40.9  [4.5] | 41.0  [4.4] | 40.8  [3.7] | | 40.7 [3.7] | 41.0  [4.4] | 41.1  [4.1] | 39.2  [4.1] | | 39.4  [3.9] | 41.4  [4.9] | 41.4  [4.4] | | 40.6  [3.8] | 40.6  [3.8] |
| **Faecal calprotectin,  µg/g** | 1963.3 [2305.2] | 1788.4 [2065.5] | | 1919.8 [2284.8] | 1955.0 [2223.0] | 1445.4 [1840.7] | 1750.4 [2182.9] | 1413.7 [1738.7] | | 1498.0 [1759.2] | 1552.2 [1968.0] | 1412.4 [1771.7] | 2097.7 [2395.6] | | 2062.3 [2263.4] | 1509.7 [1930.5] | 1730.6 [2165.9] | | 1398.1 [1728.7] | 1547.8 [1847.7] |
| **hs-CRP, mg/L** | 20.2  [35.2] | 17.8  [30.3] | | 22.2  [33.6] | 22.4  [31.6] | 16.6  [25.7] | 22.0  [31.0] | 19.8  [26.7] | | 18.6  [26.1] | 13.6 [21.7] | 12.3  [18.7] | 24.4  [37.9] | | 23.6  [34.2] | 14.8  [23.8] | 17.6  [22.4] | | 19.6  [27.0] | 21.0  [30.2] |
| **Neutrophils, %** | 70.1  [10.8] | 70.4  [9.9] | | 71.4  [9.8] | 71.9  [10.0] | 68.9  [11.1] | 70.5  [10.3] | 69.6  [10.5] | | 70.2  [10.6] | 68.8 [11.4] | 69.1  [10.2] | 71.7  [9.7] | | 72.1  [9.8] | 69.9  [11.5] | 70.9 [10.2] | | 69.1  [10.5] | 70.1  [10.7] |
| **CDAI, mean [range]** | 292.4 [220–439] | 296.3  [215–450] | | 284.7* [220–444] | 296.9  [208–447] | 285*  [220–395] | 295.7  [220–450] | 285.4* [220–439] | | 299.4  [208–447] | 287.1 [220–414] | 294.6  [220–450] | 288.8* [220–444] | | 297.4  [208–447] | 282.9* [220–391] | 297.7  [220–450] | | 286.1* [220–439] | 297.9  [208–447] |

All data are mean [standard deviation] unless otherwise specified.

*Significantly [*p* <0.05] higher prevalence of aminosalicylate use/lower CDAI value in the *TREM-1* subgroup than in the overall population.

**p* <0.05 vs overall population.

BMI, body mass index; CD, Crohn’s disease; CDAI, Crohn’s Disease Activity Index; hs-CRP, high-sensitivity C-reactive protein; *TREM-1*, triggering receptor expressed on myeloid cells.

**Supplementary Table 4.** Baseline demographics and clinical characteristics by clinical outcomes in the overall population and the subset of patients analysed for *TREM-1* expression in SERENE-CD.

| **Characteristic** | **Week 12 clinical responders** | | **Week 12 clinical non-responders** | | | **Week 56 clinical responders** | | **Week 56 clinical non-responders** | | | **Week 12 clinical remitters** | | | **Week 12 clinical non-remitters** | | **Week 56 clinical remitters** | | **Week 56 clinical non-remitters** | | |
| --- | --- | --- | --- | --- | --- | --- | --- | --- | --- | --- | --- | --- | --- | --- | --- | --- | --- | --- | --- | --- |
|  | ***TREM-1*  subset**  **[*n* = 224]** | **Overall population**  **[*n* = 421]** | | ***TREM-1* subset**  **[*n* = 42]** | **Overall population**  **[*n* = 93]** | ***TREM-1* subset**  **[*n* = 98]** | **Overall**  **population**  **[*n* = 165]** | ***TREM-1* subset**  **[*n* = 13]** | **Overall population**  **[*n* = 53]** | ***TREM-1* subset**  **[*n* = 171]** | | **Overall population**  **[*n* = 306]** | ***TREM-1* subset**  **[*n* = 95]** | | **Overall population**  **[*n* = 208]** | ***TREM-1* subset**  **[*n* = 86]** | **Overall population**  **[*n* = 140]** | | ***TREM-1* subset**  **[*n* = 25]** | **Overall population**  **[*n* = 78]** |
| **Male sex, n [%]** | 101  [45.1] | 205  [48.7] | | 20  [47.6] | 42  [45.2] | 48  [49.0] | 88  [53.3] | 5  [38.5] | 22  [41.5] | 76  [44.4] | | 141  [46.1] | 45  [47.4] | | 106  [51.0] | 44  [51.2] | 77  [55.0] | | 9  [36.0] | 33  [42.3] |
| **Age, years [range]** | 36.1  [18–73] | 36.4 [18–73] | | 37.3  [18–62] | 36.2  [18–62] | 35.3  [18–73] | 34.6 [18–73] | 39.2  [22–61] | 38.3 [19–73] | 35.3  [18–73] | | 35.7 [18–73] | 38.1  [18–67] | | 37.3  [18–67] | 34.9  [18–73] | 33.9 [18–73] | | 38.4  [20–61] | 38.5  [19–73] |
| **Disease duration, years** | 6.2  [6.8] | 7.3  [8.5] | | 6.5  [8.7] | 7.3  [8.6] | 5.3  [6.1] | 5.6  [7.2] | 12.6  [10.2] | 9.0  [9.5] | 6.0  [6.7] | | 7.0  [8.1] | 6.7  [7.8] | | 7.8  [9.1] | 5.3  [6.3] | 5.5  [7.5] | | 9.0  [8.8] | 8.0  [8.6] |
| **BMI, kg/m^2^** | 24.7  [5.8] | 24.9  [5.8] | | 24.4  [6.1] | 26.1  [7.4] | 24.1  [5.2] | 24.0 [5.5] | 25.2  [3.8] | 26.6  [6.0] | 24.4  [5.6] | | 24.5  [5.6] | 25.1  [6.2] | | 26.0  [6.7] | 24.2  [5.3] | 23.4  [4.9] | | 24.4  [4.0] | 26.8  [6.4] |
| **Corticosteroid use,**  **n [%]** | 123  [54.9] | 212  [50.4] | | 23  [54.8] | 43  [46.2] | 55  [56.1] | 90  [54.5] | 6  [46.2] | 22  [41.5] | 100  [58.5] | | 159  [52.0] | 46  [48.4] | | 96  [46.2] | 50  [58.1] | 81  [57.9] | | 11  [44.0] | 31  [39.7] |
| **Immunosuppressant use, n [%]** | 57  [25.4] | 113  [26.8] | | 8  [19.0] | 26  [28.0] | 33  [33.7] | 47  [28.5] | 1  [7.7] | 15  [28.3] | 43  [25.1] | | 81  [26.5] | 22  [23.2] | | 58  [27.9] | 28  [32.6] | 40  [28.6] | | 6  [24.0] | 22  [28.2] |
| **Aminosalicylate use,**  **n [%]** | 97*  [43.3] | 159  [37.8] | | 14  [33.3] | 23  [24.7] | 50*  [51.0] | 69  [41.8] | 7  [53.8] | 16  [30.2] | 72  [42.1] | | 115  [37.6] | 39*  [41.1] | | 67  [32.2] | 45*  [52.3] | 63  [45.0] | | 12  [48.0] | 22  [28.2] |
| **Prior infliximab use,**  **n [%]** | 34  [15.2] | 68  [16.2] | | 11  [26.2] | 21  [22.6] | 13  [13.3] | 20  [12.1] | 3  [23.1] | 10  [18.9] | 25  [14.6] | | 48  [15.7] | 20  [21.1] | | 41  [19.7] | 11  [12.8] | 17  [12.1] | | 5  [20.0] | 13  [16.7] |
| **Serum albumin, mg/mL** | 39.8  [4.2] | 39.9  [4.1] | | 39.8  [4.2] | 39.8  [4.0] | 40.8  [4.2] | 41  [4.0] | 41.4  [2.7] | 40.4  [3.9] | 39.5  [4.3] | | 39.7  [4.0] | 40.3  [4.1] | | 40.1  [4.1] | 40.7  [4.4] | 41.0  [4.1] | | 41.6  [2.4] | 40.6  [3.8] |
| **Faecal calprotectin,**  **µg/g** | 1975.4 [2362.0] | 1903.5 [2214.0] | | 1743.2 [1851.7] | 1807.9 [1883.1] | 1398.9 [1771.5] | 1648.9 [2051.1] | 998.6 [1257.8] | 1457.1 [1577.4] | 2095.6 [2344.3] | | 2019.0 [2233.8] | 1657.9 [2172.0] | | 1686.9 [2031.1] | 1454.2 [1785.1] | 1706.7 [2084.5] | | 1012.0 [1463.8] | 1416.8 [1663.2] |
| **hs-CRP, mg/L** | 21.2  [35.7] | 19.8  [31.4] | | 21.8  [25.8] | 23.6  [29.9] | 17.8  [25.5] | 21.1  [30.4] | 12.4  [14.9] | 16.7  [19.6] | 23.7  [38.7] | | 21.9  [34.3] | 16.9  [23.9] | | 18.4  [25.6] | 17.4  [25.0] | 21.6  [31.5] | | 16.5  [23.6] | 17.1  [20.8] |
| **Neutrophils, %** | 70.5  [10.4] | 71.1  [10.0] | | 72.4  [9.6] | 72.1  [9.9] | 69.2  [10.6] | 70.4  [10.6] | 66.6  [11.1] | 69.9  [10.2] | 70.1  [10.7] | | 70.8  [10.1] | 72.2  [9.5] | | 71.9  [9.9] | 68.7  [11.0] | 70.6  [10.9] | | 69.7  [9.3] | 69.8  [9.9] |
| **CDAI, mean [range]** | 290.5* [220–444] | 298.0 [208–450] | | 276.5* [220–389] | 290.3  [220–438] | 282.5* [220–398] | 298.4  [208–450] | 286.2 [224–408] | 296.1  [224–416] | 281.4* [220–444] | | 289.3  [208–450] | 300.8 [220–439] | | 307.4  [220–445] | 281.5* [220–398] | 295 [208–450] | | 288  [224–408] | 303 [224–439] |

All data are mean [standard deviation] unless otherwise specified.

**p* <0.05 vs overall population.

BMI, body mass index; CD, Crohn’s disease; CDAI, Crohn’s Disease Activity Index; hs-CRP, high-sensitivity C-reactive protein; *TREM-1*, triggering receptor expressed on myeloid cells.

**Supplementary Figure 1.** *TREM-1* expression by endoscopic outcomes^a,b^ in SERENE-UC, stratified by Week 4 drug levels [per quartile].

**
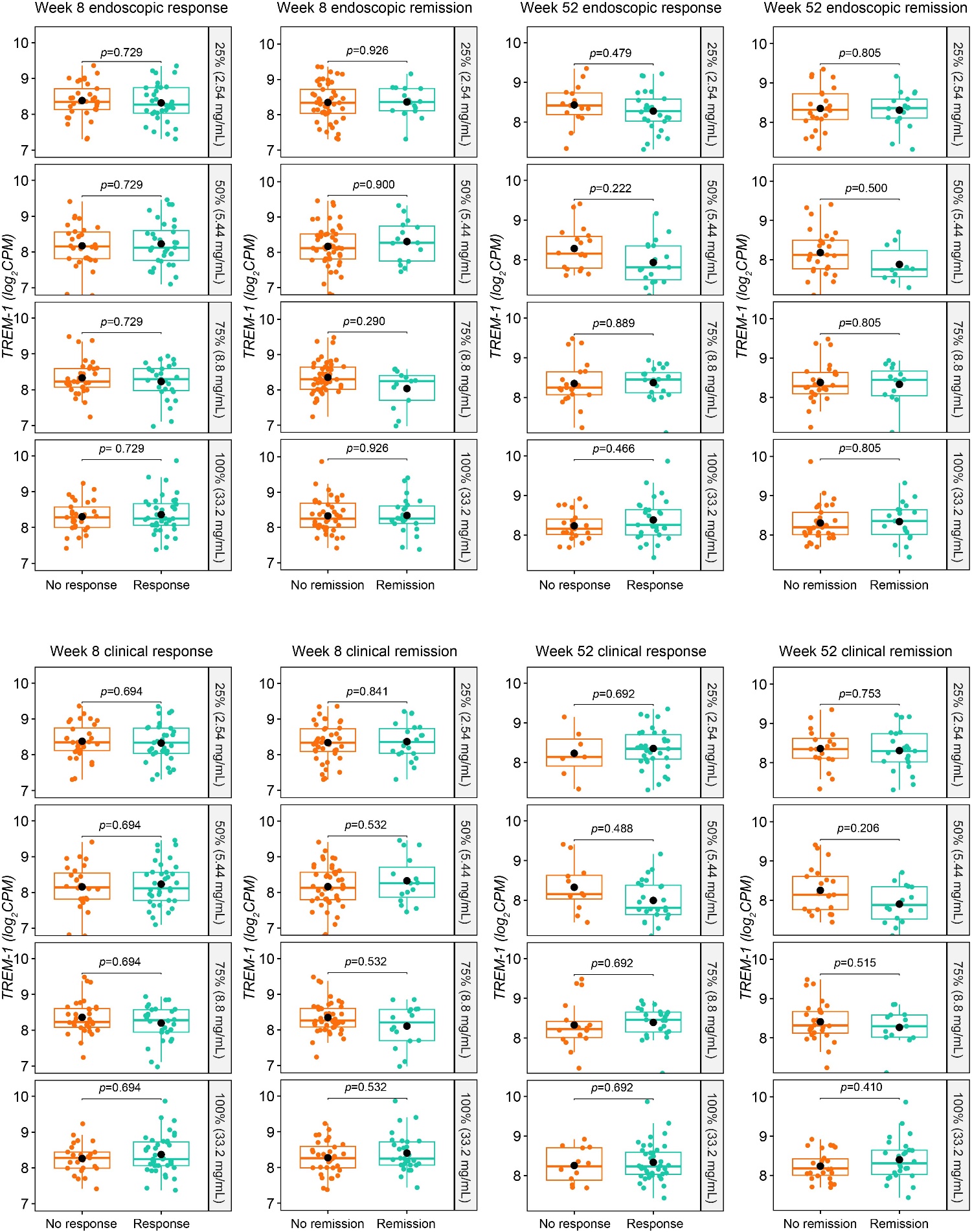
**

Data are median [interquartile range], with the p-values calculated using the baseline mean [standard deviation].

^a^Definitions of endoscopic and clinical outcomes are listed in Table 2. ^b^*n* = 95 at Week 8; *n* = 70 at Week 52.

*TREM-1*, triggering receptor expressed on myeloid cells; UC, ulcerative colitis.

**
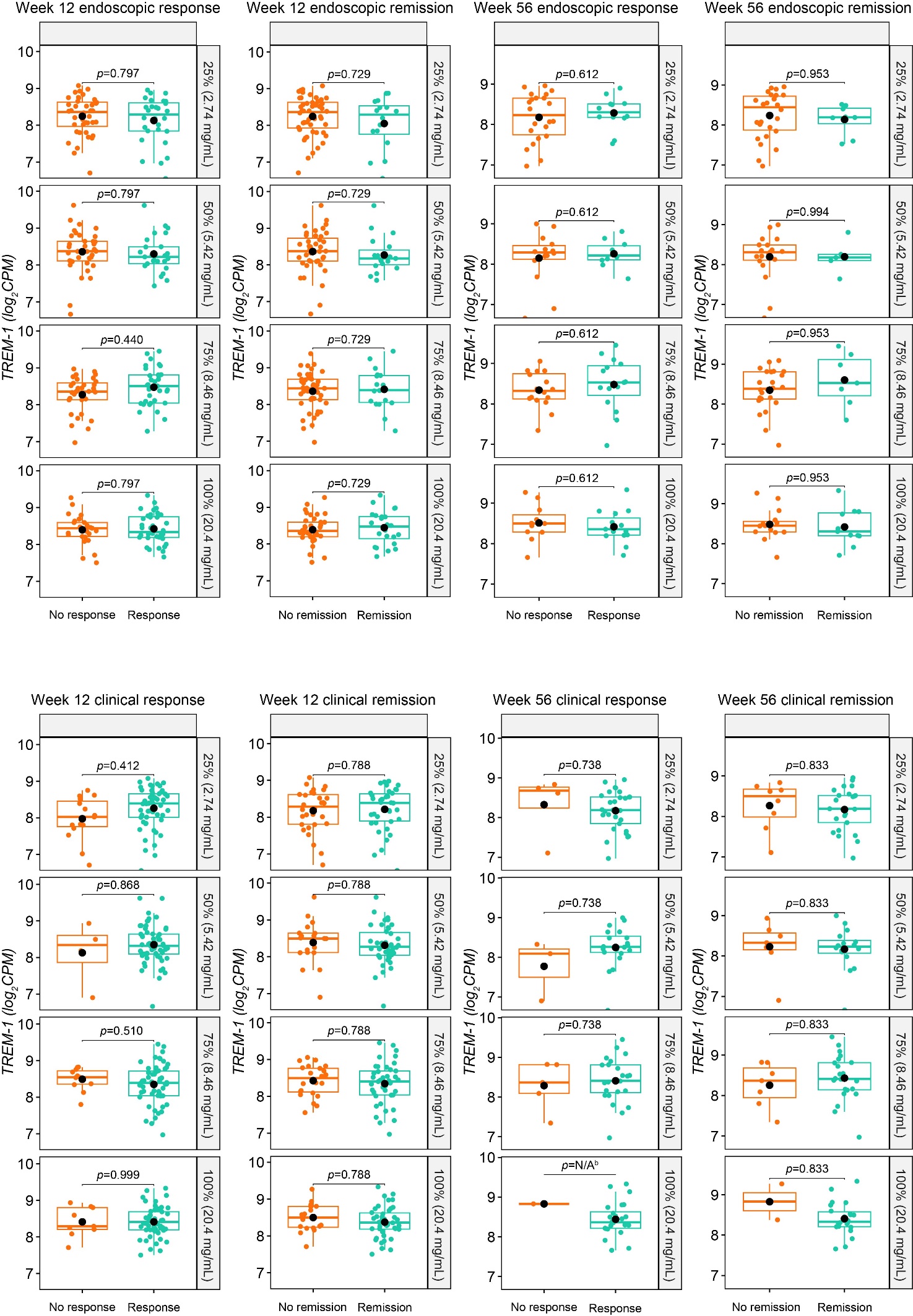
Supplementary Figure 2.** *TREM-1* expression by endoscopic outcomes^a,b^ in SERENE-CD, stratified by Week 4 drug levels [per quartile].

Data are median [interquartile range], with the p-values calculated using the baseline mean [standard deviation].

^a^Definitions of endoscopic and clinical outcomes are listed in Table 2. ^b^*n* = 106 at Week 12; *n* = 50 at Week 56.

CD, Crohn's disease; *TREM-1*, triggering receptor expressed on myeloid cells.

**Supplementary Figure 3.** *TREM-1* expression by clinical outcomes^a,b^ in SERENE-UC, stratified by Week 4 drug levels [per quartile].

**
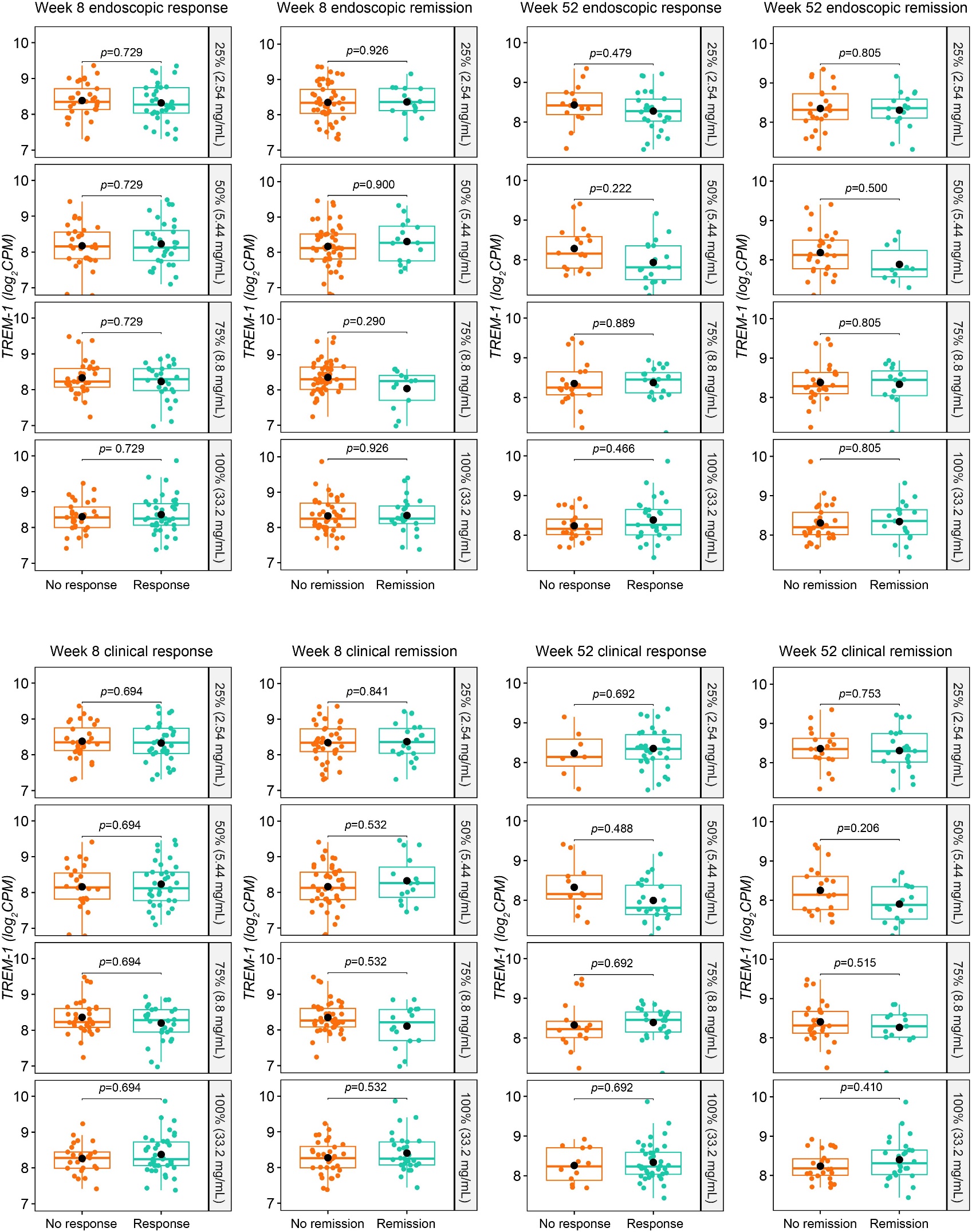
**

Data are median [interquartile range], with the p-values calculated using the baseline mean [standard deviation].

^a^Definitions of endoscopic and clinical outcomes are listed in Table 2. ^b^*n* = 95 at Week 8; *n* = 70 at Week 52.

*TREM-1*, triggering receptor expressed on myeloid cells; UC, ulcerative colitis.

**
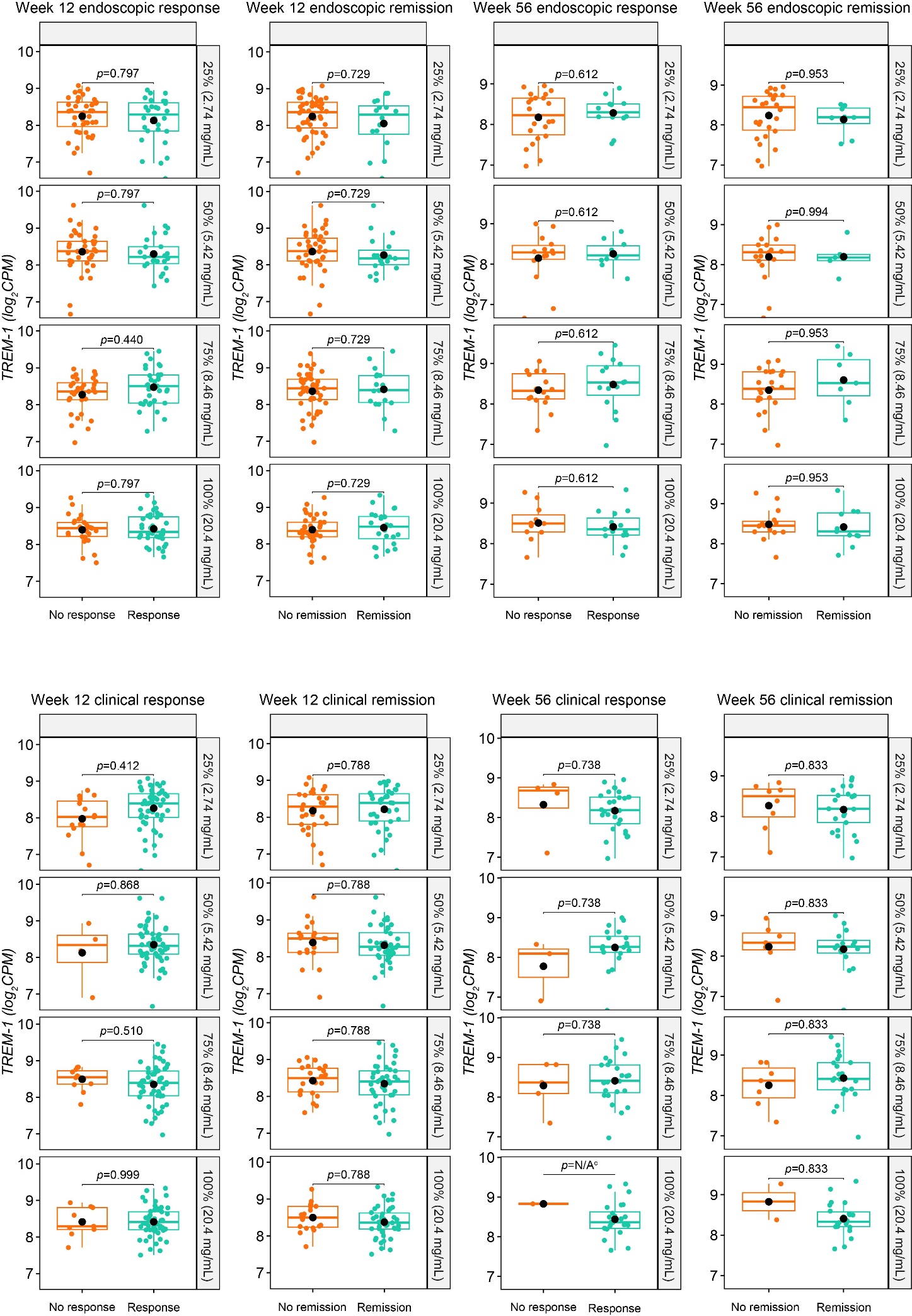
Supplementary Figure 4.** *TREM-1* expression by clinical outcomes^a,b^ in SERENE-CD, stratified by Week 4 drug levels [per quartile].

Data are median [interquartile range], with the p-values calculated using the baseline mean [standard deviation].

^a^Definitions of endoscopic and clinical outcomes are listed in Table 2. ^b^*n* = 106 at Week 12; *n* = 48 at Week 56.

^c^Only one value was available, so a statistical test could not be performed.

CD, Crohn’s disease; N/A, not applicable; *TREM-1*, triggering receptor expressed on myeloid cells.
